# Encapsulation of β-Carotene in Oil-in-Water Emulsions Containing Nanocellulose: Impact on Emulsion Properties, In Vitro Digestion, and Bioaccessibility

**DOI:** 10.3390/polym14071414

**Published:** 2022-03-30

**Authors:** Ichlasia Ainul Fitri, Wiphada Mitbumrung, Ploypailin Akanitkul, Numphung Rungraung, Varongsiri Kemsawasd, Surangna Jain, Thunnalin Winuprasith

**Affiliations:** 1 Department of Agricultural Product Technology, University of Mercu Buana Yogyakarta, Karanglo 55752, Indonesia; ichlasia@mercubuana-yogya.ac.id; 2 Institute of Nutrition, Mahidol University, Nakhon Pathom 73070, Thailand; wiphada.mit@gmail.com (W.M.); ploypailin.aka@mahidol.ac.th (P.A.); numphung.run@mahidol.ac.th (N.R.); varongsiri.kem@mahidol.ac.th (V.K.); 3 Department of Food Science and Technology, University of Tennessee, Knoxville, TN 37996, USA; surangnajn8@gmail.com

**Keywords:** encapsulation, β-carotene, emulsion, nanocellulose, fat digestion

## Abstract

The objective of this study was to explore the influence of nanocellulose type (nanocrystalline cellulose (NCC) and nanofibrillated cellulose (NFC)) and concentrations (0.05–0.20%, *w*/*w*) on the physicochemical properties, microstructure, and in vitro digestion of β-carotene loaded emulsions and β-carotene bioaccessibility. The optimum conditions for the formation of stable β-carotene loaded emulsions were found when NCC was used as a stabilizer at a concentration of 0.2% *w*/*w*. This was due to the rod-shaped structure of NCC, which led to more stable emulsions with smaller droplet size and reduced flocculation. During the in vitro gastrointestinal digestion, NFC emulsions at increased concentrations were found to retard free fatty acid (FFA) release from the emulsions and reduce the bioaccessibility of β-carotene. On the other hand, NCC emulsions at concentrations of 0.2% *w*/*w* promoted lipolysis and demonstrated highest β-carotene bioavailability. Hence, these emulsions could be used for the delivery of β-carotene with potential applications in the development of functional foods and nutraceuticals.

## 1. Introduction

Nowadays, people are focusing more on preventing non-communicable diseases (NCDs) that are the result of combinations of genetic, physiological, and environmental behaviors, especially food intake, such as high fat, sugar, and sodium intake. Hence, there has been considerable interest in the utilization of natural bioactive compounds as functional ingredients to prepare functional foods and beverages. β-carotene is one such bioactive compound that belongs to the carotenoid family and is found in many fruits and vegetables like carrots, pumpkins, mangoes, and spinach [1]. The consumption of this bioactive compound is good for human health, as it can help to decrease the risk of different chronic diseases [2]. The disease preventing activity of β-carotene could be attributed to the fact that they can be enzymatically converted to vitamin A with the conversion factor 12:1 [3]. However, β-carotene is susceptible to chemical degradation due the presence of unsaturated hydrocarbon, particularly when it is exposed to oxygen, light, and high temperatures. This results in color fading and loss of their bioactive properties [4,5]. In addition, β-carotene is a hydrophobic bioactive compound that leads to its poor water solubility, low bioavailability, and low chemical stability and restricts their incorporation into different foods and beverages [6]. Hence, to enhance its stability, solubility, and compatibility, as well as to protect this bioactive ingredient from several environmental stresses, it should be encapsulated within colloidal delivery system (CDS), such as nanoparticles assembled from food-grade biopolymers or lipids [1,7]. In addition, the encapsulation technique can also help to protect them from the harsh environment of the human gastrointestinal tract (GIT) and control their release at the desired target site [1,8,9,10].

Emulsions are one of the most common colloidal delivery systems for encapsulating hydrophobic bioactive compounds because they contain two phases (oil and water phases). This allows for more controlled and targeted release of the encapsulated bioactive compounds throughout the gastrointestinal tract (GIT) [10,11,12]. For the formation of emulsions, food grade emulsifiers such as surfactants, phospholipids, and polysaccharides are needed [13]. However, to produce emulsions that are stable to droplet coalescence, stabilizers such as polysaccharide gums and cellulose are beneficial. Each type of emulsifier and stabilizer has their own particular advantages and limitations. Therefore, it is important to use the most appropriate emulsifier and stabilizer for specific applications [12]. Nowadays, there are considerations to use natural plant-based ingredients over synthetic or animal-based food ingredients. This includes nanocellulose, where the nano size dimensions of nanocellulose can be used to enhance the encapsulation efficiency of emulsions due to higher emulsion stability. It has been reported that the more stable the emulsion, the higher is its encapsulation efficiency and lower the amount of nonencapsulated material present on particle surfaces [14]. In addition, according to the study done by Winuprasith et al. (2013), nanocellulose is a natural plant-based material that has the ability to form stable oil-in-water (O/W) emulsions through the “Pickering mechanism”, where it forms a protective steric barrier around the oil droplets and increases the viscosity of the continuous phase via the formation of a three-dimensional network [15]. Moreover, nanocellulose has attracted more attention due to its unique characteristics, such as low density, sustainability, and biodegradability [16].

Nanocellulose can be extracted from various types of agricultural by-products using chemical and mechanical methods in order to obtain nanosized (1–100 nm) and highly uniform materials [17,18]. Basically, there are three types of nanocellulose. The first is cellulose nanocrystals (CNC) or nanocrystalline cellulose (NCC), which is commonly produced by using acid hydrolysis, such as sulfuric acid. The acid hydrolyzes the amorphous regions of cellulose, thereby obtaining the crystalline regions only [19,20]. The second type is nanofibrillated cellulose (NFC) or cellulose nanofibrils (CNF), which are long entangled fibrils with diameters in the nanometer range. The NFC contains both amorphous and crystalline cellulose domains within the single fibers [21]. Nanocellulose can be categorized as a dietary fiber, particularly insoluble dietary fiber, which is poorly digested or hydrolyzed by human digestive enzymes in the gastrointestinal tract [22]. Hence, the adsorption of nanocellulose to the surfaces of lipid droplets may lead to the formation of a physical barrier that inhibits the adsorption of lipase and bile salts, and retarding lipid digestion [7,23]. The excess nanocellulose or dietary fiber in the aqueous phase of emulsions may bind with bile salts, phospholipids, or calcium, or may increase viscosity, which can also alter lipid digestion [7,24]. Due to the ability of nanocellulose to alter fat release, it should be considered that using nanocellulose as a stabilizer will have no adverse effects on the bioaccessibility of β-carotene.

The objective of the current study was to develop O/W emulsions using different types of nanocellulose (NCC and NFC) for the effective encapsulation of β-carotene. This study evaluated the effect of nanocellulose type and concentration on emulsion properties, stability, fat digestibility, and β-carotene stability and bioaccessibility. To the best of our knowledge, there have been no previous studies done where the effect of NCC and NFC as an emulsion stabilizer on the bioaccessibility of β-carotene was examined. This study has important implications for the utilization of natural plant-based stabilizers in the development of label-friendly emulsion-based delivery systems for effective encapsulation and controlled release of hydrophobic bioactive compounds.

## 2. Materials and Methods

### 2.1. Materials

Suspensions of 12.2% *w*/*w* nanocrystalline cellulose (NCC) and 3% *w*/*w* nanofibrillated cellulose (NFC) were purchased from Cellulose Lab Company, Fredericton, NB, Canada. Tween 20 and β-carotene powder (≤95% purity) were purchased from Sigma-Aldrich Company (Sigma-Aldrich, Inc., St. Louis, MO, USA). Soybean oil without any purification was purchased from a local supermarket in Nakhon Pathom, Thailand. Pepsin from porcine gastric mucosa, porcine lipase, mucin from porcine stomach, porcine bile extract, and Nile Red were purchased from Sigma-Aldrich Company (Sigma-Aldrich, Inc., St. Louis, MO, USA). Sodium azide and chloroform were purchased from Ajax Finechem (Ajax Finechem Pty., Ltd., New SouthWales, Australia). Sodium chloride, calcium chloride, monobasic phosphate, and all chemicals used in this study were of analytical grade. Double distilled water was used for the preparation of all solutions.

### 2.2. Methods

#### 2.2.1. Emulsions Preparation

An oil-in-water emulsion containing β-carotene was prepared in the dark to prevent β-carotene degradation by light. The oil phase was prepared by completely dissolving 0.005% *w*/*w* β-carotene in soybean oil, whereas the aqueous phase consisted of 1% (*w*/*w*) Tween 20 as an emulsifier, 0.01% (*w*/*w*) sodium azide as an anti-microbial agent, and 10 mM sodium phosphate buffer solution (pH = 7). A coarse emulsion was prepared by mixing 10% (*w*/*w*) oil phase and 90% (*w*/*w*) aqueous phase together using a high-shear mixer (HG-15A equipped with stator dispersing tool HT1025, Daihan Scientific Co., Ltd., Wonju, Korea) for 2 min at room temperature (25 °C). The coarse emulsion was then ultrasonicated using an ultrasonicator (Biosafer 650-92 equipped with 6 mm diameter probe, Nanjing Safer Biotech Co, Nanjing, China) for 5 min with a power rate of 50%, and pulse on/off 5 s for 5 min in order to obtain a fine emulsion. The emulsion containing β-carotene was kept at room temperature (25 °C) for 1 h to obtain the equilibrium condition. NCC or NFC were then added to the emulsion at concentrations of 0.05%, 0.10%, and 0.20% (*w*/*w*) after which the final emulsions with different concentrations of NCC and NFC were obtained after mild stirring at room temperature (25 °C) for 1 h to completely dissolve NCC and NFC in the emulsions. After emulsion preparation, the emulsions were immediately kept in an amber glass bottle overnight before further analysis.

#### 2.2.2. Visual Creaming Stability

Freshly prepared emulsions were directly transferred into a transparent glass test tube (20 mm diameter and 70 mm height) and sealed with a plastic cap. The sample tubes were kept at room temperature (25 °C) in a dark chamber, and the separation of the creaming boundary was observed for 60 days and calculated as the creaming index. The creaming index (*CI*) was measured with the equation as follows:

(1) $$CI\left(\%\right)=\left(\frac{H_{S}}{H_{T}}\right)\times 100$$

where $H_{T}$ is total height of the emulsion, and $H_{S}$ is serum phase height.

#### 2.2.3. Particle Size Measurement

The particle size and size distribution of the freshly prepared emulsions and digested emulsions were analyzed by a laser diffraction particle size analyzer (Mastersizer 2000, Malvern Instruments Ltd., Worcestershire, United Kingdom). The samples were diluted in 10 mM phosphate buffer solution (pH = 7) to avoid multiple scattering effects. The refractive indices of the oil and water phases used in the calculations were 1.46 and 1.33, respectively. Particle diameter was reported as the surface-weighted mean diameter (*d*_32_), which was calculated from the full particle size distribution.

#### 2.2.4. ζ-Potential Measurement

Particle charges of the droplets in the freshly prepared emulsions and digested emulsion were measured using a particle electrophoresis instrument (Zetasizer Nano ZS, Malvern Instruments Ltd., Worcestershire, United Kingdom). The samples were diluted using 10 mM phosphate buffer solution (pH = 7) to avoid multiple scattering effects. The particle charge was reported as the average and standard deviation of measurements made on three freshly prepared samples, with two readings taken per sample.

#### 2.2.5. Apparent Viscosity Measurement

Viscosity of the freshly prepared emulsions was measured using a controlled-strain rheometer (Physica MCR 301, Anton Paar GmbH., Graz, Austria) equipped with a cone and plate sensor (1° cone angle, 50 mm diameter, and 0.05 mm gap). The viscosity information was obtained from steady flow tests. The measuring sensor was programmed to linearly increase the shear rate from 0.1 s^−1^ until 300 s^−1^ in 3 min followed immediately by a reduction from 300 s^−1^ to 0.1 s^−1^ in the next 3 min. The temperature of measurement was controlled at 25 °C.

#### 2.2.6. Quantification of β-Carotene Content

The β-carotene content in the emulsions was measured using the method described previously with some modifications in a dark room [4]. In brief, the β-carotene in emulsion samples were extracted by using chloroform and then centrifuged at 1,600 rpm for 10 min (TGL-16B, Anting Inc., Shanghai, China). The transparent lower organic phase containing β-carotene was collected and then quantified using an UV–Vis spectrophotometer (UV-1601 SHIMAZU, Shimadzu co., Kyoto, Japan) at 450 nm. The concentration of β-carotene was then determined using a standard calibration curve (R^2^ = 0.990). The encapsulation efficiency (*EE*) of the emulsions was calculated using the equation as follows:

(2) $$EE\left(\%\right)=\frac{\beta -carotenecontentpresentinsample}{Amountof\beta -caroteneaddedinitialy}\times 100$$

#### 2.2.7. Confocal Laser Scanning Microscopy (CLSM)

The microstructure of the emulsions was analyzed using confocal laser scanning microscopy (CLSM) supported by an objective lens (NikonD-Eclipse C1 80i, Nikon Inc., Melville, NY, USA). The oil phase of the emulsions was stained using Nile Red dye (1 mg/mL ethanol). An aliquot of the stained sample was put into microscope slide and then covered with a cover slip. The excitation and emission spectra for Nile Red were 543 nm and 605 nm, respectively. As Nile Red stained the oil phase, individual oil droplets and regions rich in lipids appeared as bright patches, whereas the aqueous phase appeared as dark regions in the micrographs.

#### 2.2.8. In Vitro Digestion Model

The freshly prepared emulsions were diluted in 10 mM phosphate buffer solution (pH = 7) to achieve the required oil concentration of 2% *w*/*w*. These samples were then passed through a multi-stage static gastrointestinal tract (GIT) model, including mouth, stomach, and small intestinal phase [25]. Every GIT stage was performed in the dark room to prevent β-carotene degradation induced by light. The digestion procedure used was recently updated INFOGEST digestion stimulation protocol [26].

Initial system: 20 mL of the diluted samples was put into a 100 mL glass beaker and incubated in a water bath (model Memmert WNE 7, Memmert Co., Island, Skandinavia) at 37 °C for 2 min.

Mouth stage: The simulated saliva fluid (SSF) containing 0.03 g/mL mucin was preheated to 37 °C for 3 min. Then, 20 mL of SSF was mixed with the initial emulsion and its pH adjusted to 6.8. The mixture was then swirled continuously in a water bath (model Memmert WNE 7, Memmert co., Island, Skandinavia) at 37 °C for 2 min to mimic agitation conditions in the mouth.

Stomach stage: Simulated gastric fluid (SGF) was prepared by dissolving 2 g of sodium chloride (NaCl) and 7 mL of hydrochloric (HCl) in double distilled water. The total volume of SGF was then adjusted to 1 L. The sample (20 mL) from the mouth phase was mixed with 20 mL of SGF containing 0.0032 g/mL pepsin in a beaker. Then, the pH was adjusted to pH = 2.5 and swirled continuously in a water bath (model Memmert WNE 7, Memmert Co., Island, Skandinavia) at 37 °C for 2 h to mimic the conditions in the stomach.

Small intestine stage: 20 mL of sample from the stomach phase was transferred into a beaker and put into a water bath at 37 °C, which was connected to an automatic titration unit used as a pH-stat (Metrohm, USA Inc., Riverview, United States). The pH was adjusted to pH = 7 using an NaOH solution. Simulated intestinal fluids (1.5 mL) containing 0.25 M CaCl_2_ and 3.75 M NaCl were added into the reaction vessel, after which 3.5 mL of bile salt solution (5 mg/mL) was added in the system with continuous stirring. The pH was adjusted back to pH = 7 using NaOH. Freshly prepared 2.5 mL lipase solution (1.6 mg/mL) was added immediately to the reaction, and then the automatic titration unit started. The titration unit was used to monitor and maintain the pH at 7 by titrating 0.10 M NaOH solution into the reaction vessel for 2 h. The temperature was controlled at 37 °C during the experiment. The number of moles of NaOH is 0.10, which is required to neutralize the free fatty acids (FFAs). The FFA released was then calculated according to the equation described:

(3) $$\%FFA=\frac{V_{NaOH}\times m_{NaOH}\times M_{Lipid}}{W_{Lipid}\times 2}$$

Here *V_NaOH_* is the volume of NaOH required to neutralize the FFAs produced (in L), *m_NaOH_* is the molarity of the NaOH solution (0.10 M), *W_Lipid_* is the total weight of lipid initially present in the reaction vessel (0.25 g), and *M_Lipid_* is the molecular weight of the oil used (920). After each stage of GIT, the samples were collected and measured for particle size, particle charge, confocal microscopy, β-carotene stability, and bioaccessibility.

#### 2.2.9. β-Carotene Bioaccessibility

At the end of the GIT model, the fresh digesta (from small intestine) was centrifuged at 4000 rpm for 45 min (TGL-16B, Anting Inc., Shanghai, China), and then the bottom micelle phase was collected as raw digesta. The extraction method of β-carotene was based on previous studies with some modifications [27]. The measurement was performed in the dark room. The chloroform was added to the raw digesta and centrifuged at 6,500 rpm for 10 min to obtain the mixed micelle. The bottom layer as an extracted β-carotene was collected and then analyzed by a UV–Vis spectrophotometer (UV-1601 SHIMAZU, Shimadzu Co., Kyoto, Japan) at 450 nm. The concentrations of β-carotene were determined using a pre-prepared calibration curve. The bioaccessibility of β-carotene and stability of β-carotene were calculated according to the equation described in previous studies.

(4) $$Bioaccessibility\left(\%\right)=\left(\frac{C_{Micelle}}{C_{Digesta}}\right)\times 100$$

(5) $$Stability\left(\%\right)=\left(\frac{C_{Digesta}}{C_{DInitial}}\right)\times 100$$

Here, $C_{Micelle}$ is the concentration of β-carotene in the mixed micelle phase, $C_{DInitial}$ is the concentration of β-carotene in the emulsion, and $C_{Digesta}$ is the concentration of β-carotene in the total digesta collected after the small intestine phase.

#### 2.2.10. Statistical Analysis

All measurements were performed in triplicate. The data were reported as mean ± standard deviation. A one-way analysis of variance (ANOVA) with Duncan’s multiple range test was used to indicate significant differences at *p* < 0.05. The statistical analysis was performed using the commercial software (SPSS Inc., Chicago, IL, USA).

## 3. Results

### 3.1. Influence of Nanocellulose on the Properties and Stability of Emulsions Containing β-Carotene

The influences of nanocrystalline cellulose (NCC) and nanofibrillated cellulose (NFC) at different concentrations on the encapsulation efficiency (*EE*) of β-carotene in emulsions are shown in Table 1. It was observed that the EE of the control emulsions was significantly lower (*p* < 0.05) than those of the emulsions containing nanocellulose. This suggests that the addition of NCC and NFC increased the *EE* of β-carotene in the emulsions. However, no significant (*p* ≥ 0.05) differences were observed in the *EE* of β-carotene between emulsions containing NCC and NFC at different concentrations. The higher *EE* value of emulsions containing NCC and NFC suggests that the emulsions were more stable, and there was a reduced number of β-carotene at the oil surface and higher retention of the active material [28]. Moreover, Rein et al. (2012) and Costa et al. (2019) suggested that both NCC and NFC have the ability to form stable O/W emulsions due to the fact that they are wetted better in water than oil solution, which can increase the stability of emulsions for further applications [29,30].

**Figure 1:**
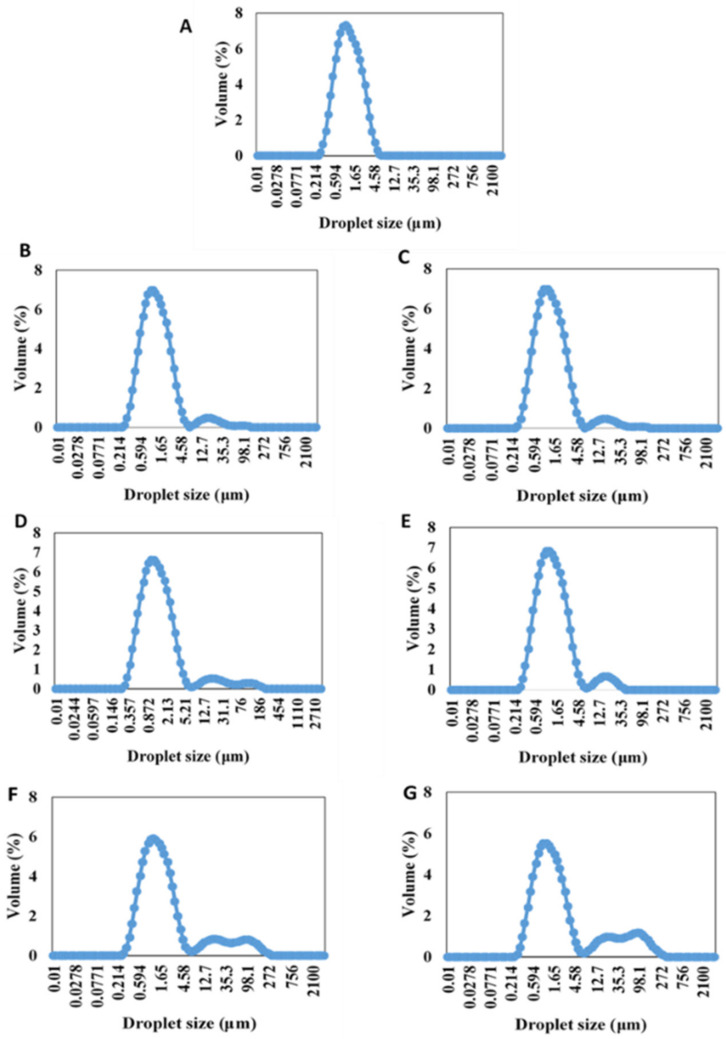
Particle size distributions of 10% (*w*/*w*) O/W emulsions containing β-carotene without nanocellulose (**A**) and with nanocellulose, including 0.05% (*w*/*w*) NCC (**B**), 0.10% (*w*/*w*) NCC (**C**), 0.20% (*w*/*w*) NCC (**D**), 0.05% (*w*/*w*) NFC (**E**), 0.10% (*w*/*w*) NFC (**F**), and 0.20% (*w*/*w*) NFC (**G**).

The particle size and size distribution of the emulsions containing β-carotene are shown in Table 1 and Figure 1, respectively. There were no significant differences (*p* ≥ 0.05) observed in the *d*_32_ values of the control emulsion and the emulsions containing NCC at all concentrations. However, the emulsions containing NFC demonstrated significantly (*p* < 0.05) bigger particle size than the others, but there were no significant (*p* ≥ 0.05) differences in particle size of NFC emulsions at different concentrations. The differences in particle size of the emulsions containing NCC and NFC can be attributed to bridging flocculation that occurred in the emulsions containing NFC [7]. The difference in effect between NCC and NFC on particle size of emulsions could also be explained in terms of their structure. The structure of native NCC is short and rod shaped [31], which can be easily dispersed in the emulsions. On the other hand, the structure of NFC is comprised of a long entanglement fibril structure that acts as a bridge between oil droplets, thereby inducing oil droplet flocculation [32]. This phenomenon can be explained in term of a stabilization effect by increasing the viscosity of the continuous phase, as shown in Table 1, by the three-dimensional network of NFC, thereby preventing coalescence of oil droplets, which was beneficial for long-term storage of the emulsion [33,34]. In addition, the depletion mechanism may also be another reason that can explain the NFC-induced flocculation phenomena. The NFC particles are excluded from a narrow region surrounding each lipid droplet, thereby generating an osmotic attraction that forces the droplets together [7].

ζ-potential represents the surface charge of the oil droplets, which is expressed in terms of the electrokinetic potential of the colloidal systems that helps to determine emulsion stability. A higher magnitude of ζ-potential indicates higher electrostatic repulsion between the oil droplets that provides greater stability to the emulsion systems. The low negativity charge in the control emulsion could be because this emulsion consisted of only Tween 20, which is a non-ionic surfactant. The magnitudes of ζ-potential of the emulsions containing either NCC or NFC were higher than the control (Table 1). It can be explained as follows: nanocellulose is an anionic polysaccharide so some of them may accumulate at the oil surface, thereby increasing the magnitude of ζ-potential. The correlation between ζ-potential and emulsion stability could be confirmed by the creaming measurement.

**Figure 2:**
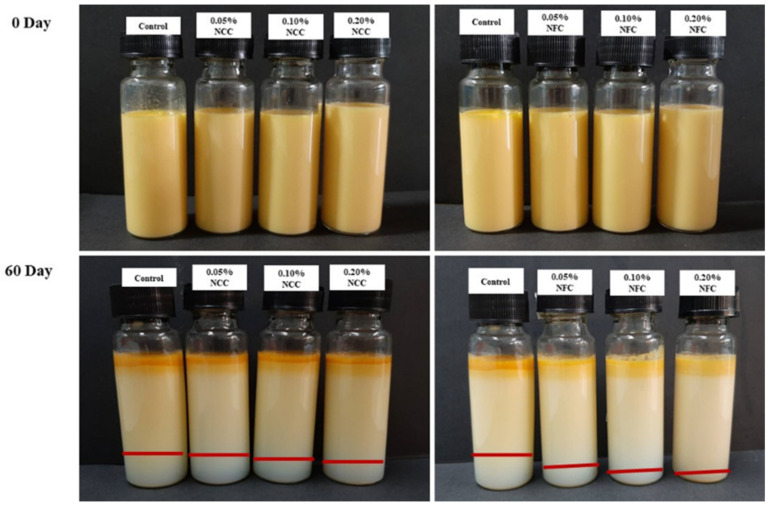
Appearance of 10% O/W emulsions containing β-carotene stabilized by NCC or NFC at various concentrations at 0.05, 0.10, and 0.20% *w*/*w* at 0 day and 60 days of storage time at room temperature (25 °C).

Phase separation of emulsions after storage for 60 days is clearly seen in Figure 2, especially in emulsions-stabilized by NCC. For the determination of creaming index (Table 1), the control emulsion had the highest creaming index (21.70%). It was clearly observed that the lower creaming indices were found in the emulsions containing nanocellulose. Further, the creaming indices decreased when NCC and NFC concentrations were increased. Interestingly, the lowest creaming index was found in the emulsions containing 0.20% NFC (4.65%), which can be explained by the viscosity effect of NFC. This result suggests that the viscosity of the emulsion was strongly dependent on the NFC concentration due to the existing three-dimensional network structures at the continuous phase that helps to prevent the droplets from aggregation and coalescence [33,34]. McClements (2015) mentioned Stokes’ law, which suggests that the higher viscosity of the continuous phase could be used to prevent creaming [35]. In addition, delayed creaming could be attributed to the depletion flocculated network, which was formed in the presence of non-adsorbing nanocellulose particles [16].

### 3.2. Influence of Nanocellulose on Gastrointestinal Fate of Lipid Droplets

The mean particle diameter, ζ-potential, and microstructure of 10% (*w*/*w*) O/W emulsion prepared using Tween 20 as an emulsifier or NCC or NFC as a stabilizer were measured after passing through the different stages of the simulated gastrointestinal tract (GIT).

#### 3.2.1. Initial Stage

**Figure 3:**
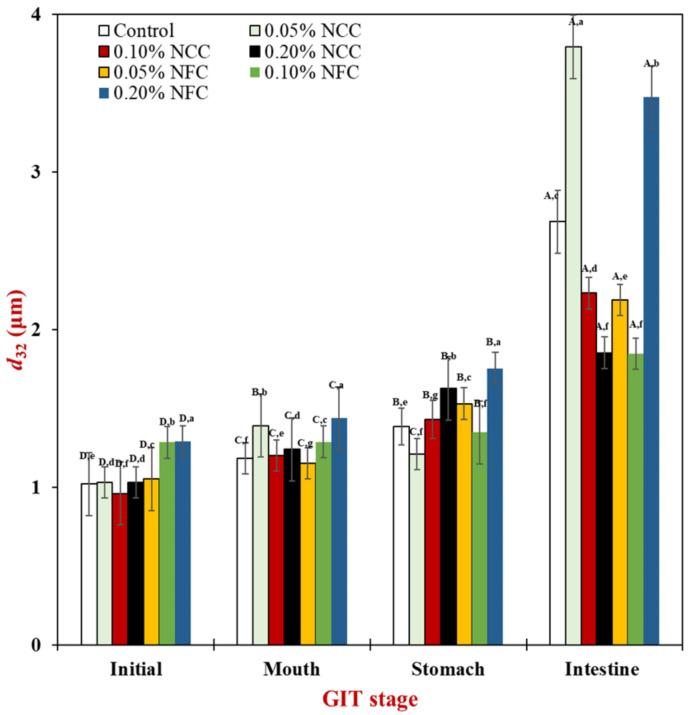
Influence of nanocellulose and gastrointestinal tract (GIT) stage on mean droplet diameter of 10% (*w*/*w*) O/W emulsions containing β-carotene. Samples designated with different capital letters (A−D) were significantly different (Duncan, *p* < 0.05) when compared between different GIT stages (same stabilizer). Samples designated with different lowercase letters (a−g) were significantly different (Duncan, *p* < 0.05) when compared between different stabilizer (same GIT stage).

Firstly, the mean particle diameter, ζ-potential, and microstructure of emulsions before entering the GIT were observed to identify the stability of emulsions after 24 h storage. The control emulsions without nanocellulose demonstrated significantly smaller surface-weighted mean particle diameter (*d*_32_) than the NFC-stabilized emulsions. Moreover, it was observed that when 0.20% (*w*/*w*) NCC was added to the emulsions, it led a decrease in the mean particle diameter of emulsions (Figure 3). On the other hand, the *d*_32_ of emulsions stabilized with NFC was found to significantly increase (*p* < 0.05) with increasing concentration of NFC. This suggests that NCC was more effective in producing smaller particle sizes than NFC. The different stabilization mechanism of NCC and NFC to stabilize the emulsions can be attributed to their different physical properties. According to Kalashnikova et al. (2013), NCC has a rod shape structure due to which it can easily disperse in the emulsion system [31]. Further, NCC has been successfully used to produce smaller oil droplet size (below 250 nm) in emulsions in comparison to when NFC is used as an emulsifier. Therefore, it may help to prevent flocculation by the good dispersion of NCC in the continuous phase due to the short length structure of NCC.

**Figure 4:**
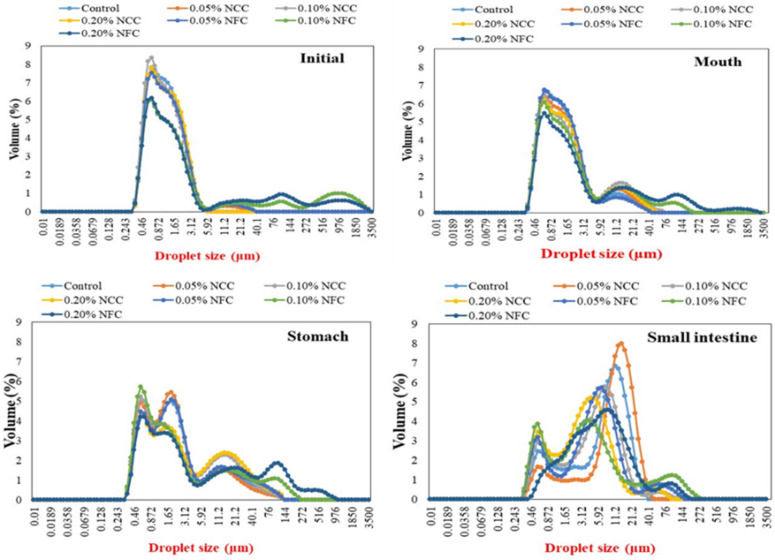
Influence of nanocellulose and gastrointestinal tract (GIT) stage on particle size distribution of 10% (*w*/*w*) O/W emulsions containing β-carotene.

In contrast, NFC has long flexible fiber networks that can achieve extended-chain conformations in the continuous phase, which can influence the continuous phase and the emulsion characteristics (e.g., viscosity) [36]. Moreover, it also leads to the formation of porous and multilayered organizations, thereby acting as a bridge between oil droplets, which is called the bridging flocculation phenomenon [7]. This can be confirmed by the particle size distribution data, where it was observed that the emulsions stabilized by NFC exhibited multimodal distribution with more than one single peak (Figure 4). Moreover, when the concentration of NFC was increased, they exhibited a wider particle size distribution than the emulsions stabilized by NCC. Multimodal distribution with more than one single peak is often encountered in food emulsions due to flocculation [35]. The flocculation that occurred in the emulsions stabilized by NFC was further confirmed by confocal microscopy, where larger clusters of flocs were observed in the emulsions with increasing NFC concentration, which appeared as large regions of red color.

**Figure 5:**
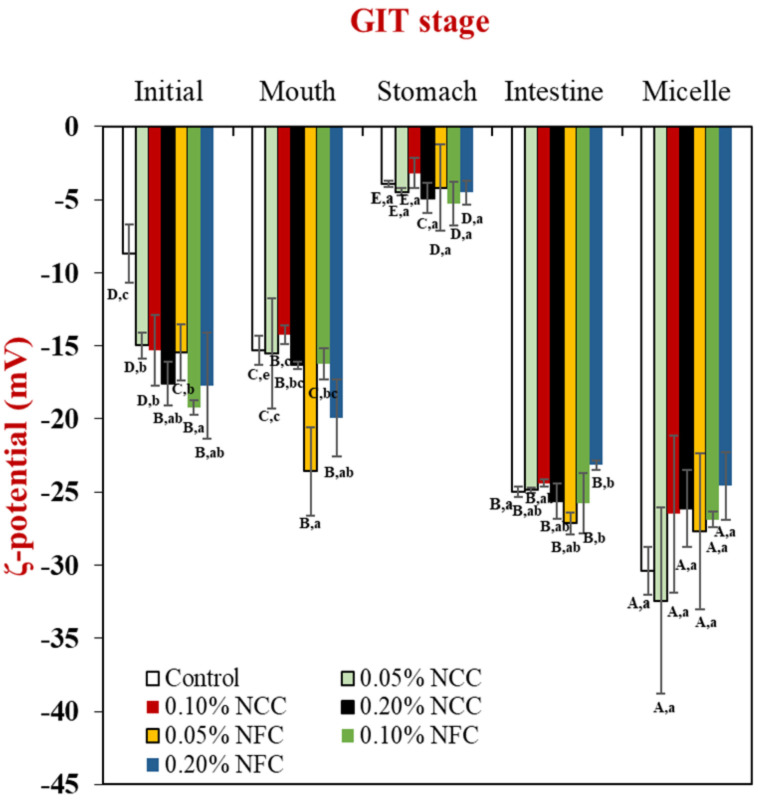
Influence of nanocellulose and gastrointestinal tract (GIT) stage on ζ-potential of 10% (*w*/*w*) O/W emulsions containing β-carotene. Samples designated with different capital letters (A−E) were significantly different (Duncan, *p* < 0.05) when compared between different GIT stages (same stabilizer). Samples designated with different lowercase letters (a−c, e) were significantly different (Duncan, *p* < 0.05) when compared between different stabilizer (same GIT stage).

The influence of nanocellulose type and concentration on ζ-potential of the initial O/W emulsions is shown in Figure 5. The magnitude of ζ-potential of the control emulsion without nanocellulose addition was significantly lower (*p <* 0.05) than those of the emulsions containing either NCC or NFC. The low negativity charge in the control emulsion could be because this emulsion consisted of only Tween 20, which is a non-ionic surfactant [37]. In fact, the non-ionic surfactant would not be expected to give any charge. Nevertheless, it has been shown that the control emulsion had an appreciable negative charge, which had been attributed to preferential adsorption of hydroxyl ions (OH−) from the aqueous phase or the presence of anionic impurities during the emulsion preparation from Tween 20 or oil used, such as fatty acids [35].

**Figure 6:**
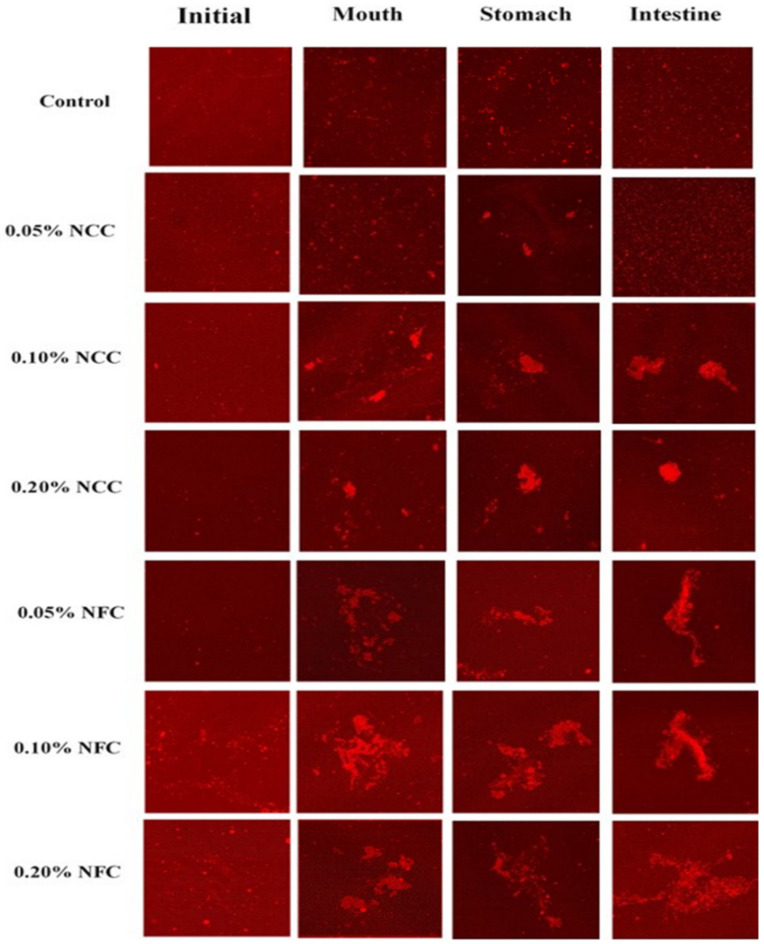
Influence of nanocellulose and gastrointestinal tract (GIT) stage on microstructure of 10% (*w*/*w*) O/W emulsions containing β-carotene at scale bar 50 μm.

The ζ-potentials of the initial stage of emulsions containing NCC or NFC were higher than −15 mV. The NCC and NFC are anionic polysaccharides that promote electrostatic repulsive forces by NCC and NFC itself. Based on our observations, aqueous NCC and NFC itself promoted higher negativity charge (20–25 mV). In addition, nanocellulose may be located between oil droplets that generate the repulsive forces between nanocellulose itself and oil droplets. It is also supported by Mitbumrung et al. (2019) that addition of NFC helped to increase the ζ-potential (>25 mV) of the emulsions [38]. In addition, the presence of sulfuric acid during extraction of NCC exhibited the surface OH^−^ groups of NCC that partially react with the acid, creating charged sulfate ester groups on its surface that leads to the high magnitude of ζ-potential [39]. The higher negativity charge (Figure 5) and large cluster of oil droplets (Figure 6) induced by the three-dimensional structure of NFC promoted more stable emulsions with less phase separation than the control and the emulsions stabilized by NCC. This phenomenon can be explained by several factors, especially due to bridging flocculation phenomenon and high viscosity that helps to prevent the drop droplet movement, resulting in higher stability of emulsions containing NFC [7,33].

#### 3.2.2. Mouth Stage

After exposure to the mouth stage, the mean particle diameter (Figure 3) in all tested emulsions slightly increased from around 0.90–1.00 μm to 1.20–1.50 μm, especially by the addition of NCC at concentrations less than 0.20% (*w*/*w*) and NFC at concentrations more than 0.05 (*w*/*w*), respectively, as seen by the multimodal distribution data (Figure 4) in all tested emulsions. The confocal laser scanning micrographs showed larger clusters of lipid droplets in the emulsion containing nanocellulose than the control emulsion without nanocellulose (Figure 6). Moreover, the cluster of oil droplets were found to be held together by the fiber, as the concentration of NCC and NFC increased from 0.05 to 0.20% (*w*/*w*). This phenomenon was more prominent in the emulsion stabilized by NFC. NFC had diameters in the nanometer range but lengths of several micrometers or more and provided its functionality by forming three-dimensional networks in the continuous aqueous phase. The network of aggregated NFC fibers may have adsorbed to the surfaces of two or more droplets simultaneously, thereby causing them to be held together in a cluster [7,16]. An increase in the *d*_32_ values of all emulsions was observed after exposure to the mouth stage. It has been suggested that small flocculation, especially bridging or depletion flocculation, of the lipid droplets can take place due to the presence of mucin in the saliva [40]. Depletion flocculation is known to occur due to the presence of relatively high amounts of non-adsorbed anionic mucin in the aqueous phase that increases the osmotic attraction between the lipid droplets [41,42]. A slight increase in the ζ-potential (Figure 5) of all emulsions was also observed after exposure to the mouth phase, which can be attributed to adsorption of anionic species (such as mucin) from the simulated saliva to the droplet surfaces [43].

#### 3.2.3. Stomach Stage

After passing through the stomach phase, the mean particle diameter of all tested emulsions was found to increase significantly (*p* < 0.05) (except 0.05% (*w*/*w*) NCC emulsions) (Figure 3). The increase in NCC and NFC concentration led to an increase in the droplet size of emulsions, which could be confirmed by evidence from particle size distribution showing multimodal distributions (Figure 4). The confocal laser micrographs (Figure 6) demonstrated that the oil droplets were highly flocculated for all tested emulsion samples, especially the emulsions containing nanocellulose at high concentrations. The increase in particle size and presence of flocs can be explained by several factors. Firstly, the gastrointestinal environment condition changed from neutral to acidic when the emulsions passed from the mouth stage to the stomach stage, leading to an alteration in the surface charge of Tween 20-coated oil droplets. Secondly, electrostatic screening by counter-ions in the simulated gastric fluid (SGF) decreased the electrostatic repulsion between the droplets. Thirdly, mucin from simulated saliva may cause bridging flocculation, leading to flocs and an increase in particle size [44]. When comparing NCC and NFC emulsions, it was observed that NFC-stabilized emulsions demonstrated bigger particle size than the NCC-stabilized emulsions as concentration increased from 0.05% to 0.20% (*w*/*w*). NCC emulsions at concentrations higher than 0.05% (*w*/*w*) and NFC emulsions at all concentration remained as relatively large cluster lipid droplets and were held together by the fiber. The ζ-potentials of all emulsions were found to decrease steeply, as shown in Figure 5, after exposure to the stomach phase because the low pH and high ionic strength in simulated gastric fluid may reduce the ionization of the anionic charged groups [9].

#### 3.2.4. Small Intestinal Stage

There was a significant increase (*p* < 0.05) in the mean particle diameter of all tested samples after exposure to the small intestine phase (Figure 3). On examination of the full particle size distribution of the emulsions, it was seen that they exhibited the multimodal distribution (Figure 4) with more than two peaks with more irregular shape than at the previous stage, indicating that these droplets were unstable to aggregation. The confocal images (Figure 6) showed that the control emulsion contained much finer droplets than the others due to the fact that fat digestion began at the small intestine with the removal of some oil phase from emulsions leading to a loss of their dimension [7]. For emulsions stabilized by 0.05% (*w*/*w*) of NCC, there appeared to be a fair distribution of lipid rich particles throughout the confocal images. The single droplet seemed bigger than control emulsions and emulsions made using other concentrations of NCC. However, as NCC concentration was increased from 0.10% (*w*/*w*) to 0.20% (*w*/*w*), the emulsions exhibited less cluster, and it was difficult to distinguish the individual droplets or small flocs due to the limitation of resolution of the confocal microscope. The bright red color in the confocal image is the lipid rich zone, which may have consisted of undigested oil. On the other hand, as the concentration of NFC increased from 0.05% to 0.20% (*w*/*w*), there was a larger cluster of the lipid rich zones remaining in the images, which may due to the fact that there was more insoluble matter remaining in this system, especially oil droplets connected with nanocellulose.

Interestingly, the light scattering data show that the largest mean particle diameter was present for emulsions containing 0.05% (*w*/*w*) of NCC and the smallest was present for emulsions containing 0.20% (*w*/*w*) of NCC or 0.10% (*w*/*w*) of NFC. However, the confocal images suggested that the largest clusters of oil droplets present in the NFC stabilized emulsions was when the concentration increased from 0.05% (*w*/*w*) to 0.20% (*w*/*w*). These differences may have been due to the analytical methods used to determine the microstructure of the sample. For the particle size measured by the laser diffraction instrument, the agitation protocol was applied during measurement, thereby destroying the flocs or oil clusters. However, both measurements (particle size and confocal microscopy) are always used to characterize the emulsion structure to confirm or distinguish the microstructure of the emulsion whether it contains floc or it is homogeneous.

The ζ-potential of all emulsions increased steeply, as shown in Figure 5, which can be attributed to several factors. First, the pH in the small intestine increased, which promoted the higher ζ-potential in the emulsions. Second, anionic bile salt and phospholipid from the bile extract tend to absorb into the lipid surface due to which there was an increase in the negative charge [45,46]. Third, anionic free fatty acids generated from the digestion of triglycerides promoted the higher negativity charge. From the observation of mixed micelle centrifuged from raw digesta, the negativity charge was found to be extremely high, which can be attributed to the presence of FFA and bile salt [8].

#### 3.2.5. Influence of Nanocellulose on Fat Digestibility

An automatic titration method was used to identify the extent of fat digestion in the emulsions as potential delivery systems with different types and concentrations of nanocellulose. The free fatty acid released from the sample during the digestion was calculated based on the volume of sodium hydroxide that was titrated to keep the pH of the sample at a constant value (pH = 7).

**Figure 7:**
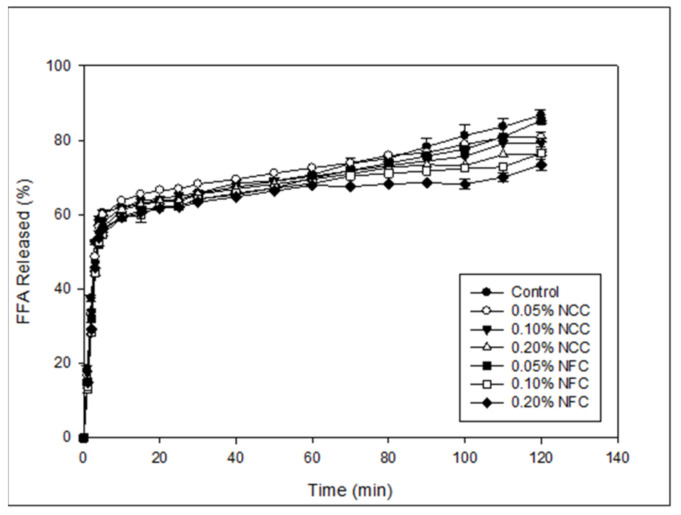
Influence of nanocellulose type and concentration on the release of free fatty acids (FFA) from 10% (*w*/*w*) O/W emulsions containing β-carotene under simulated small intestinal conditions.

**Figure 8:**
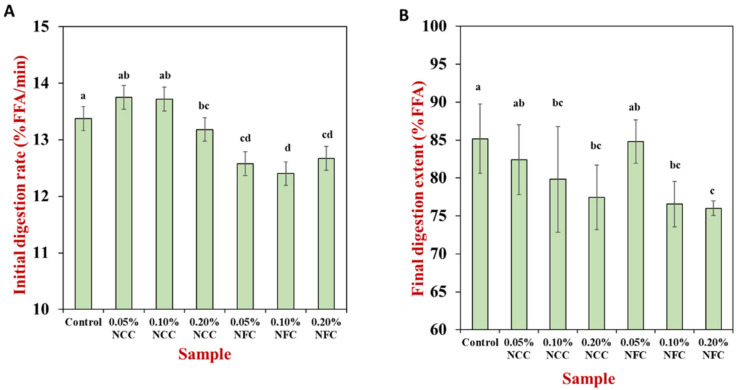
Initial digestion rate (**A**)and final digestion extent (**B**) of 10% (*w*/*w*) O/W emulsions containing β-carotene stabilized by either nanocrystalline cellulose (NCC) or nanofibrillated cellulose (NFC) at various concentrations. Samples designated with different lowercase letters (a−d) were significantly different (Duncan, *p* < 0.05).

The initial rate and final extent of free fatty acid release were measured from the data, where the initial rate was measured by fitting a straight line to the first five minutes of digestion, and the final extent was calculated from the average of the last five minutes of digestion. Generally, there was a rapid increase in the FFA release after the first 5–10 min of digestion due to the presence of the free lipid droplets, after which the FFA release remained relatively constant (Figure 7). The fastest initial digestion rate and the highest final extent of FFA release (Figure 8) were found in the control emulsion that did not contain nanocellulose. The reduced initial digestion rate was observed in the emulsions containing nanocellulose, which was more prominent in the emulsion containing 0.20% (*w*/*w*) of NCC and emulsions containing NFC at all concentration. For the final extent of lipid digestion, the highest FFA release was found in the control, 0.05% (*w*/*w*) of NCC, and 0.05% (*w*/*w*) of NFC emulsions. This effect can be attributed to the nanocellulose concentration. At lower concentrations, nanocellulose was not enough to inhibit or retard the FFA release due to less aggregation of oil droplets in the emulsions, especially at the initial until stomach phase of GIT. However, the increase in NCC and NFC concentrations higher than 0.05% (*w*/*w*) led to a decrease in the final extent of lipid digestion. It was also observed that 0.20% NFC was the most effective nanocellulose type and concentration to decrease the rate of FFA release.

The rate of lipid digestion was strongly dependent on the size of the lipid droplets. The control, 0.05% (*w*/*w*) NCC, and 0.05% (*w*/*w*) NFC emulsions had small mean droplet diameter in the initial phase and was less aggregated at stomach phase when compared with the other emulsions, which led to the greater interaction between surface area of lipid and lipase, leading to the higher digestion rate and extent [42]. The emulsions stabilized by 0.20% (*w*/*w*) NCC had more droplet aggregation than 0.05% (*w*/*w*) NCC and 0.10% (*w*/*w*) NCC-stabilized emulsions at the initial and stomach phase, thereby leading to the release of smaller amounts of FFA due to the minimal surface area of oil droplets that were exposed to the digestion fluid.

The emulsion stabilized by NFC, except for 0.05% (*w*/*w*) of NFC, exhibited the slower rate of initial digestion and lower final digestion extent because of the bigger mean particle diameter and large cluster of oil droplets at the stomach phase. This led to a decrease in the surface area of oil droplets that were exposed to the lipase enzyme. Moreover, the increase in concentration of nanocellulose decreased the rate of initial digestion and final digestion extent. The slower digestion rate can be explained by several factors. First, addition of NCC and NFC to the emulsion can increase the viscosity of the emulsion. This is especially the case for NFC, as NFC has a long entanglement structure with interconnected fiber that can prevent access between lipase and the oil surface. In this case, the lipase could not move easily to the oil droplets because of higher emulsion viscosity. Second, the increase in concentration of NFC caused an increase in the particle size of emulsions and increased the larger clusters of oil droplets, thereby decreasing the surface area of oil droplets available for lipolysis [44]. Third, NCC and NFC are categorized as anionic polysaccharides [26] that may bind with the calcium ion to remove digested lipid from the lipid droplet. However, the final digestion extent of 0.05% (*w*/*w*) NFC emulsion was not significantly different from the control and 0.05% (*w*/*w*) NCC emulsions even though it had higher viscosity than the control. This can be attributed to the fact that the microstructure of 0.05% (*w*/*w*) of NFC was less aggregated in comparison to the other concentrations of NFC, especially at the initial and stomach phase (Figure 6).

#### 3.2.6. Influence of Nanocellulose on Bioaccessibility and Stability of β-carotene

**Figure 9:**
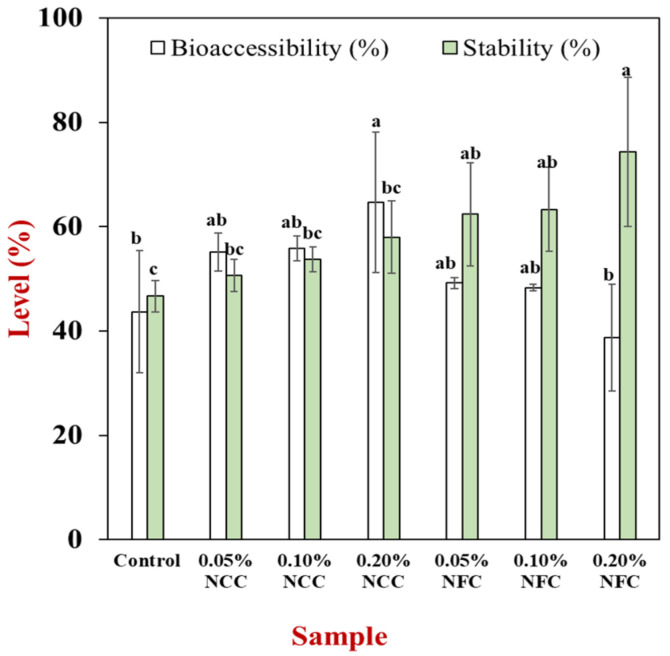
Influence of nanocellulose type and concentration on β-carotene stability and β-carotene bioaccessibility of 10% (*w*/*w*) O/W emulsions containing β-carotene stabilized by either nanocrystalline cellulose or nanofibrillated cellulose (NFC) at various concentrations. Samples designated with different lowercase letters (a−c) were significantly different (Duncan, *p* < 0.05).

β-carotene remaining in both raw digesta and mixed micelle were studied after their passage through the small intestine. The initial amount of β-carotene was 0.005 g of β-carotene in 100 g of sample. After exposure through the GIT (raw digesta), the bioacessibility of β-carotene was measured by comparing with the β-carotene content at the micelle. Based on Figure 9, there was an increase in the bioaccessibility of β-carotene of the emulsion stabilized by NCC at all concentrations and NFC at concentrations lower than 0.20% (*w*/*w*). However, the most effective type and concentration of nanocellulose to increase the bioaccessibility of β-carotene was 0.20% (*w*/*w*) of NCC. Generally, a comparison between β-carotene bioaccessibility is dependent on the total amount of FFA released at the end of GIT, where β-carotene bioaccessibility is higher when the amount of FFA released is higher. Further, addition of NCC at concentrations from 0.05% to 0.20% (*w*/*w*) led to an increase in the bioaccessibility of β-carotene, whereas increasing the concentration of NFC from 0.05 to 0.20% (*w*/*w*) led to a decrease in the β-carotene bioaccessibility. However, it was observed that at 0.20% NCC, the β-carotene bioaccessibility was the highest, which could be attributed to the fact that these emulsions had finer single lipid droplets (Figure 6), and the cluster was assumed to be a mixed micelle present, which could solubilize the β-carotene.

Generally, smaller particle size, i.e., higher surface area, improves the oil digestibility and transfer of β-carotene to micelles [47]. It is further supported that the particle size had more impact on carotenoid bioaccessibility than cell wall presence, with smaller particle size and more fine chewing significantly enhancing bioaccessibility, presumably due to enhanced access of digestion enzymes [48]. In line with these results, emulsions with small droplet diameters (0.2–23 µm) improved β-carotene transfer from lipid droplets to mixed micelles and bioaccessibility from approximately 35–60% [44]. The emulsion stabilized by NFC did not have any effect on bioaccessibility of β-carotene, which correlates with the reduced amount of FFA released from emulsions stabilized by NFC at concentrations from 0.05% to 0.20% (*w*/*w*). Moreover, the emulsions stabilized by NFC at concentrations higher than 0.05% (*w*/*w*) promoted bridging flocculation due to the long entanglement fiber that led to lesser amounts of FFA released, which may be the cause of lower β-carotene bioaccessibility.

Furthermore, to understand things more deeply, β-carotene stability was examined (Figure 9). The stability of β-carotene was calculated by comparing the amount of β-carotene that remained at the small intestine (raw digesta) in comparison to the original amount. It was observed that the stability of β-carotene in emulsions without addition of NCC and NFC was the least due to the higher surface area with less aggregation. On the other hand, the stability of β-carotene in the emulsions stabilized by NCC and NFC increased, especially at concentration of 0.20% (*w*/*w*) of NFC. This can be due to the fact that the higher NFC concentration helped to protect β-carotene from inappropriate conditions in GIT, especially in the stomach, resulting in an increase of its remaining content [49]. Moreover, it may relate with the properties of emulsions containing NFC at the GIT tract. As mentioned previously, the bigger oil droplet clusters in the emulsion containing nanocellulose promoted higher viscosity compared with the control emulsion. The small surface area of the emulsions stabilized by NFC had less contact with environmental stresses, especially at the stomach phase [50].

## 4. Conclusions

This study has provided important information for the development of β-carotene emulsions using nanocellulose as potential delivery systems to enhance β-carotene stability and bioaccessibility after passing through the simulated gastrointestinal tract. It was observed that by modulating the nanocellulose type and concentration, the digestive rate of fat and β-carotene bioaccessibility could be controlled. Emulsion with higher NCC concentration or lower NFC concentration could help to increase β-carotene stability and bioaccessibility. This finding is a promising strategy that can be used to develop functional foods and nutraceuticals using nanocellulose as a stabilizer to modulate fat digestibility, control release, as well as promote β-carotene stability and bioaccessibility. However, in the future it would be useful to study the in vivo gastrointestinal fate of this type of delivery system for a better understanding.

## Figures and Tables

**Table 1:** Encapsulation efficiency (*EE*), surface-weighted mean particle diameter (*d*_32_), apparent viscosity at shear rate of 100 s^−1^ (*Ƞ*_a,100_), ζ-potential, and creaming index after a storage period of 60 days of the 10% (*w*/*w*) O/W emulsions containing β-carotene stabilized by NCC or NFC at various concentrations at 0.05, 0.10, and 0.20% (*w*/*w*).

Emulsion Sample	Nanocellulose Concentration (%)	*EE* (%)	*d_32_*(μm)	*Ƞ*_a,100_ (Pa.s)	ζ-Potential (mV)	Creaming Index (%)
Control	0	96.60 ± 0.98 b	0.98 ± 0.01 b	0.00126 ± 0.001 a	−8.69 ± 1.99 c	21.70 ± 0.02 a
NCC	0.05	99.88 ± 0.01 a	0.97 ± 0.02 b	0.00128 ± 0.001 a	−14.97 ± 0.90 b	13.80 ± 0.01 b
	0.10	99.83 ± 0.59 a	1.03 ± 0.03 b	0.00130 ± 0.001 a	−15.30 ± 2.43 b	13.60 ± 0.72 b
	0.20	99.65 ± 0.01 a	0.99 ± 0.03 b	0.00138 ± 0.001 a	−17.57 ± 1.50 ab	12.60 ± 0.02 b
NFC	0.05	99.74 ± 0.06 a	1.39 ± 0.01 a	0.0042 ± 0.001 a	−15.46 ± 1.94 b	11.59 ± 0.01 b
	0.10	99.73 ± 0.06 a	1.35 ± 0.01 a	0.0087 ± 0.001 a	−19.73 ± 0.50 a	9.72 ± 0.46 c
	0.20	99.71 ± 0.01 a	1.37 ± 0.01 a	0.5016 ± 0.152 b	−17.73 ± 3.63 ab	4.65 ± 0.30 c

Note: Assays were performed in triplicate. Mean ± standard deviation values in the same column were followed by different lowercase letters (a−c) showing a significant difference (*p* < 0.05).

## Data Availability

Not applicable.
